# Cerebellar Ataxia Caused by Type II Unipolar Brush Cell Dysfunction in the Asic5 Knockout Mouse

**DOI:** 10.1038/s41598-020-58901-y

**Published:** 2020-02-07

**Authors:** Tabita Kreko-Pierce, Nina Boiko, Donald G. Harbidge, Daniel C. Marcus, James D. Stockand, Jason R. Pugh

**Affiliations:** 1 Department of Cellular and Integrative Physiology, University of Texas Health Science Center at San Antonio, San Antonio, TX 78299 USA; 2 Department of Anatomy and Physiology, Kansas State University, Manhattan, KS 66506 USA

**Keywords:** Ion channels in the nervous system, Cerebellum

## Abstract

Unipolar brush cells (UBCs) are excitatory granular layer interneurons in the vestibulocerebellum. Here we assessed motor coordination and balance to investigate if deletion of acid-sensing ion channel 5 (Asic5), which is richly expressed in type II UBCs, is sufficient to cause ataxia. The possible cellular mechanism underpinning ataxia in this global Asic5 knockout model was elaborated using brain slice electrophysiology. Asic5 deletion impaired motor performance and decreased intrinsic UBC excitability, reducing spontaneous action potential firing by slowing maximum depolarization rate. Reduced intrinsic excitability in UBCs was partially compensated by suppression of the magnitude and duration of delayed hyperpolarizing K^+^ currents triggered by glutamate. Glutamate typically stimulates burst firing subsequent to this hyperpolarization in normal type II UBCs. Burst firing frequency was elevated in knockout type II UBCs because it was initiated from a more depolarized potential compared to normal cells. Findings indicate that Asic5 is important for type II UBC activity and that loss of Asic5 contributes to impaired movement, likely, at least in part, due to altered temporal processing of vestibular input.

## Introduction

The cerebellum plays a key role in the control of balance, and motor coordination and precision. In this regard, the cerebellum integrates multisensory inputs to fine-tune motor activity, and dysfunction of the cerebellum causes ataxia^1–3^. The vestibulocerebellum comprised of the flocculonodular lobe, including lobules IX and X, integrates, in particular, vestibular information to influence balance and muscle tone.

The basic neural circuit is highly regular throughout the cerebellum, including excitatory afferent mossy fiber (MF) inputs that synapse on granule cells (GCs) with GCs sending excitatory parallel fibers to Purkinje cells (PCs). One exception to this regular pattern is the unipolar brush cell (UBC) found chiefly in the vestibulocerebellum and dorsal cochlear nucleus (DCN)^4^. Like GCs, UBCs are excitatory glutamatergic interneurons receiving synaptic input from MFs, but UBCs synapse onto other cells in the granule cell layer instead of PCs^4–7^. The function of UBCs and the reason for their nearly exclusive expression in the granule cell layer of the vestibulocerebellum is obscure.

Differences in lobular distribution, size, abundance, and histochemical and electrical properties have led to further subdivision of UBCs into two types. Type I UBCs typically have larger somata and rich expression of calretinin and PLCβ1^8^. In comparison, type II UBCs are relatively smaller and robustly express mGlurR1α and PLCβ4^7,8^. The majority, about 2/3, of UBCs are type II. Type I UBCs are primarily found in the dorsal section of the granular layer of lobules X and IXd^7,8^. Type II UBCs, in contrast, have the richest expression in the ventral granular layer of lobule X with notable expression also in all areas of lobule IX, excluding IXa, with some expression also in the vermis of lobule VIII^6,8^. Such differences in expression patterns may reflect afferent-specificity being related to terminal fields of different vestibular MFs. It also could reflect differences in UBC subtype function.

The two types of UBCs have different electrophysiological properties particularly in response to glutamate^6,9^. Type II UBCs are less prone to fire spontaneously, and are excited by glutamate with burst firing due to high expression of AMPARs and mGluR1α, and little mGluR2-mediated GIRK current. In contrast, type I UBCs typically fire spontaneously at relatively high frequencies and glutamate provokes an inhibitory response. This inhibitory response in type I UBCs results from little AMPAR-mediated current combined with robust outward K^+^ currents activated by mGluR2. Through analogy to the retina, such observations have refined understanding of UBC function: In response to MF glutamatergic transmission, type II UBCs may function as ON cells that upregulate spiking following a brief phase delay; and, type I UBCs may function as OFF cells with downregulated firing in response to glutamate resulting in a phase inversion. Together these two types of UBCs allow distinct parallel processing of their inputs to their target GCs resulting in diverse phase coding capable of enhancing adaptive control of behavior by facilitating appropriate output in a broad temporal window^5,6,9–11^.

Abnormal UBC function, similar to the dysfunction of other key cerebellar neurons, may contribute to ataxia^3,12–14^. For instance, the reduced number and misplacement of UBCs in the *reeler* mouse is thought to be an early contributor to the ataxic phenotype of this model; though, the *reeler* mouse has generalized cerebellar neuronal wasting and misplacement^15^. Dysfunction and ultimately ablation of type II UBCs is also thought to make a primary contribution to the ataxic phenotype of the *Moonwalker* mouse; though, *Trpc3*, the defective gene in this model, is also expressed and essential for normal PC function and health^1–3,14,16^. Because the mouse models that have implicated UBC dysfunction in ataxia generally have wider cerebellar neural dysfunction and loss, it has been difficult to determine with certainty if UBC dysfunction alone is capable of causing ataxia.

The acid-sensing ion channels (Asics) are ligand-gated ion channels in the Degenerin/ENaC channel family that conduct depolarizing inward Na^+^ currents^17–22^. This is an ancient ion channel family expressed in every metazoan species^23,24^. Asics are almost exclusively expressed in neurons of the central and peripheral nervous systems. Mammals express five Asic genes, *Accn1-5*. Compared to Asic1-4, little is known about Asci5, encoded by *Accn5*, which also has been called BLiNaC, hINaC and BASIC, previously^25–27^. Asic5 is unique for an Asic channel in that it is expressed in both neurons of the CNS and epithelial cells in the liver, albeit, with very restricted expression in each of these tissues^25–27^. In liver cells, Asic5 is known to be activated by bile acids^25,28^; however, in neurons the endogenous ligand has not yet been identified. As we have demonstrated previously, Asic5 is restrictively expressed, in the brain, in type II UBCs^29^. The functional significance of Asic5 expression in type II UBCs remains obscure.

The current studies were designed to test the importance of Asic5 to type II UBC function and to test whether targeted dysfunction of type II UBCs was capable of causing ataxia. Tests of motor coordination and balance combined with electrophysiological analysis of type II UBCs in an Asic5 knockout mouse demonstrated that targeted disruption of normal type II UBC function was capable of causing ataxia. The underlying pathophysiological mechanism, as well as cellular compensation associated with the ataxic phenotype in the Asic5 knockout mouse, also were revealed. Loss-of-function of Asic5 decreases intrinsic excitability of type II UBCs by slowing the rise time of the action potential resulting in a decrease in spontaneous spiking. This decrease in intrinsic excitability was partially compensated by suppression of the magnitude and duration of hyperpolarizing K^+^ currents stimulated by glutamate. Consequently, type II UBCs lacking Asic5 have decreased intrinsic spiking, and a reduced magnitude and duration of hyperpolarization following glutamate stimulation resulting in elevated burst firing. This likely causes abnormal temporal processing in cerebellar circuits containing these interneurons leading to the motor discoordination and balance deficits observed in the Asic5 KO mouse.

## Results

### Asic5 is expressed in type II unipolar brush cells

**Figure 1:**
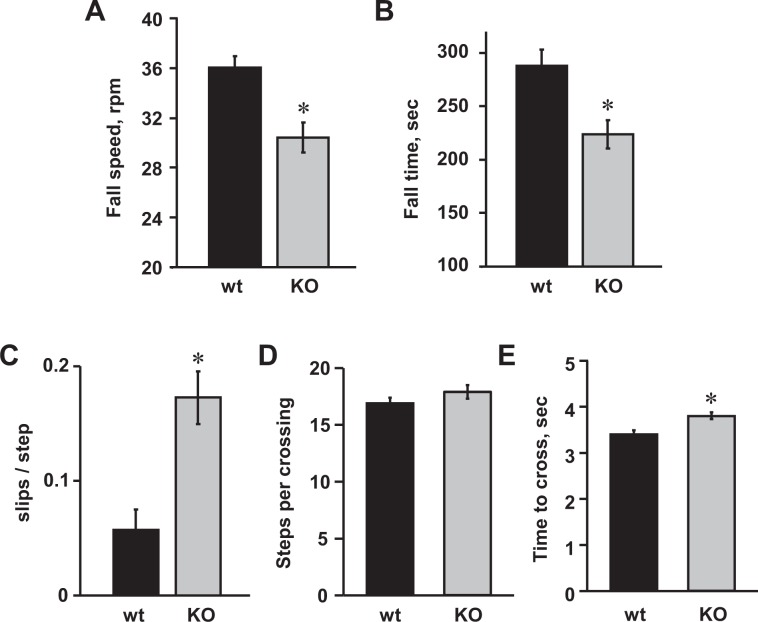
Mature Asic5 KO mice exhibit ataxic behavior. Summary graphs of mean fall speeds (**A**) and fall times (**B**) for trained mature wild type (black bars) and Asic5 KO (gray bars) mice as quantified using an accelerating rotarod test. Balance beam test: Summary graphs comparing the mean number of slips per step taken (**C**), steps taken per crossing (**D**), and total time taken to cross (**E**) for trained mature littermate control (black bars) and Asic5 KO (gray bars) mice during a full run on an elevated horizontal balance beam. Summary data for both experiments from 10 different male littermate (83.0 ± 3.0 days old, 27.1 ± 0.5 g) and 10 different male Asic5 KO (90.5 ± 0.5 days, 25.8 ± 0.6 g) mice where each mouse was tested once in triplicate. *Significantly different using a two tailed unpaired *t*-test.

We began the current studies by confirming an earlier observation made in the *Asic5*^*tm2a(KOMP)Wtsi*^ reporter mouse that Asic5 is restrictively expressed in type II UBCs of the vestibulocerebellum^29^. β-Galactosidase (β-Gal) expression is driven by the *Asic5* promoter in this reporter mouse. Supplemenary Fig. 1 contains a representative fluorescence micrograph of lobules IX and X in a midsagittal section from the cerebellum of the *Asic5*^*tm2a(KOMP)Wtsi*^ reporter mouse. This representative cerebellum section was immuno-stained with an anti-β-Gal antibody (red) and co-stained with DAPI (blue). β-gal expression in the brains of the Asic5 reporter mouse was restricted to granular layer interneurons within lobules X, IXb and IXc, but not IXa. As shown by the representative β-gal positive interneuron magnified in the inset, β-gal positive cerebellar interneurons have hallmark features of UBCs: A rounded cell soma bearing a cardinal dendrite that ramifies into a brush-like tuft of short dendrioles. This is consistent with selective expression of Asic5 in UBCs and our previous findings showing Asic5 expression only in mGluR1α positive cells, a marker of type II UBCs^29^.

### The Asic5 knockout mouse

To explore the function of Asic5 and type II UBCs, we created an Asic5 knockout mouse from the Asic5^*tm2a(KOMP)Wtsi*^ reporter mouse used in this earlier work^29^. Supplementary Fig. 2A shows a simplified scheme describing creation of this knockout mouse. The SA-βgeo-pA gene trapping cassette between exons 2 and 3 in the reporter allele was removed first with FLP-mediated recombination producing the floxed mouse. The *Asic5* gene was then disrupted at exon 3 through Cre-mediated recombination to produce the global knockout. Standard PCRs were used to confirm proper FLP-FRT and Cre-LoxP recombination and dependent generation of floxed and KO mice. As shown in Supplementary Fig. 2B,C, unique primer combinations capable of discriminating between the wild type, floxed and KO alleles produced the expected PCR products in genotyping reactions on homozygous wild type, floxed and Asic5 KO mice, respectively.

### Asic5 KO mice have impaired motor coordination

Restricted expression of Asic5 in type II UBCs suggests that this channel may play a role in the physiology of the vestibulocerebellum. We tested this possibility by comparing the balance and motor coordination of homozygous Asic5 KO with that of littermate control mice. As shown in Fig. 1A,B, mature Asic5 KO mice were significantly impaired in their ability to run on an accelerating rotarod, falling sooner than wild type mice (223.9 ± 13.1 vs. 287.8 ± 15.4 sec, respectively) and at slower speeds (30.4 ± 1.2 vs. 36.02 ± 0.94 rpm, respectively). Similarly, as shown in Fig. 1C, mature Asic5 KO mice performed less well compared to littermate controls on an elevated horizontal balance beam making significantly more slips per step in a single crossing, approximately 3 fold more than control mice (0.17 ± 0.02 vs. 0.06 ± 0.01 slips/step, respectively). Control and knockout mice, as shown in 1D, used a similar number of steps to cross the balance beam (16.9 ± 0.05 vs. 17.9 ± 0.6 steps, respectively), but knockout compared to control mice, as shown in Fig. 1E, completed the apparatus significantly more slowly (3.8 ± 0.08 vs. 3.4 ± 0.09 seconds, respectively). These results demonstrate that deletion of Asic5 diminishes balance and motor coordination in mice when normal balance and movement is challenged. Such an observation is consistent with impaired cerebellar function.

### Impaired motor coordination in Asic5 KO mice is present early in life

**Figure 2:**
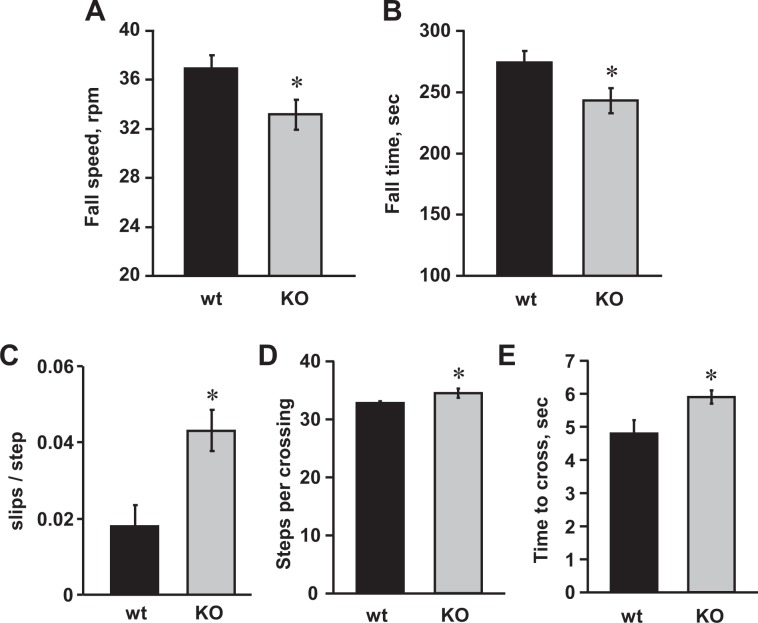
Weanling Asic5 KO mice exhibit ataxic behavior. Summary graphs of mean fall speeds (**A**) and fall times (**B**) for trained wild type (black bars) and Asic5 KO (gray bars) mice as quantified using an accelerating rotarod test. Summary data from 12 different male littermate (24.9 ± 0.2 days old, 11.2 ± 0.6 g) and 15 different male Asic5 KO (24.8 ± 0.4 days old, 11.7 ± 0.4) mice where each mouse was tested once in triplicate. Balance beam test: Summary graphs comparing the mean number of slips per step taken (**C**), steps taken per crossing (**D**), and total time taken to cross (**E**) for trained littermate control (black bars) and Asic5 KO (gray bars) mice during a full run on an elevated horizontal balance beam. Summary data from 13 different male littermate (28.2 ± 1.2 days old, 14.3 ± 0.8 g) and 14 different male Asic5 KO (27.8 ± 1.8 days old, 14.3 ± 1.2 g) mice where each mouse was tested once in triplicate. *Significantly different using a two tailed unpaired *t*-test.

Because the Asic5 KO mouse is a global knockout with germline transmission, we were curious to test whether the discoordination they showed as adults was also apparent earlier in life. Most animal models of inheritable ataxia have a phenotype that gets more severe with age as cerebellar neurons parish^1–3,14–16^. We find no evidence that deletion of Asic5 affects survival of type II UBCs (see below and ref. ^29^). As shown in Fig. 2A,B, weanling Asic5 KO mice, like their older brethren, were significantly impaired in their ability to run on an accelerating rotarod, falling sooner than wild type mice (242.9 ± 10.9 vs. 274.3 ± 8.9 sec, respectively) and at slower speeds (33.2 ± 1.2 vs. 36.9 ± 1.1 rpm, respectively). Similarly, as shown in Fig. 2C, weanling Asic5 KO mice performed less well compared to littermate controls on the elevated horizontal balance beam making significantly more slips per step in a single crossing (0.04 ± 0.005 vs. 0.02 ± 0.005 slips/step, respectively). Knockout compared to control mice, as shown in Fig. 2D,E, also took significantly more steps (17.9 ± 0.06 vs. 16.9 ± 0.5 steps, respectively) and time (5.9 ± 0.2 vs. 4.8 ± 0.4 seconds, respectively) to cross the balance beam. These results demonstrate that the discoordination observed in Asic5 KO mice is present early in life, and, at least as manifested on the accelerating rotarod, is of equal magnitude in weanling and mature animals. While weanling knockout mice performed less well on the balance beam compared to control littermates, both cohorts of weanlings, irrespective of genotype, performed much better than their aged peers. Thus, a common confound related to aging, independent of genotype, possibly weight, size or strength vs. mass, obscures a direct comparison between young and old mice when assessing magnitude of discoordination with performance on the balance beam. Nevertheless, both weanlings and mature Asic5 KO mice qualitatively performed less well compared to their control littermates on this apparatus.

### Unchallenged Asic5 KO mice have a normal gait

One manifestation of severe ataxia is an abnormal gait when walking normal^30^. Such abnormal gaits are hallmarks of the *Moonwalker* and *reeler* mouse models of cerebellar dysfunciton^14,15^. As shown by the gait analysis reported in Supplementary Fig. 3, mature Asic5 KO mice do not differ from control littermates in this regard. Thus, Asic5 KO mice display discoordination when challenged with difficult apparatuses that require enhanced coordination but appear normal with respect to gait (and general activity, see directly below) when unstressed. Such findings argue that the discoordination observed in the Asic5 KO mouse model represents a mild form of ataxia in comparison to that seen in the *Moonwalker* and *reeler* mouse. Alternatively, while Asic5 has rich expression in type II UBCs of the vestibulocerebellum and DCN, and almost no expression outside of these regions within the brain^29^, it is possible that this discoordination in the global Asic5 KO mouse is a manifestation of something other than a mild form of cerebellar ataxia. One possibility could be that deletion of Asic5 causes an anxiety-like phenotype, which may have affected performance on the rotarod and balance beam. While possible, the fact that no evidence supports Asic5 expression in areas of the brain implicated in anxiety-like behaviors, to include the broader forebrain, as well as prelimibic inputs to the amygdala and the amygdala itself^27,29^, seems to lessen this latter alternative explanation of discoordination in the Asic5 KO mouse.

### Asic5 KO mice have normal activity and display no anxiety-like behavior

While we think unlikely to explain the discoordination documented above in the Asic5 KO mouse, it was important to test if these mice had any anxiety-like behavior. Because the open-field test is commonly used to assay anxiety-like behaviors and also quantifies activity in unchallenged animals^31–33^, we tested performance of weanling Asic5 KO and littermate controls on this apparatus. As reported in Supplementary Fig. 4, weanling Asic5 KO mice performed no different in the open-field test compared to littermate controls with both occupying border areas approximately 25-fold longer than the central area. Moreover, distance traveled, which is reflective of normal activity, was not different in knockout versus control mice.

### Type II UBC number, morphology and capacitance are normal in Asic5 KO mice

The Asic5 KO line deletes Asic5 from all tissues, raising the possibility that motor coordination defects are due to gross changes in cerebellar development or morphology. However, we found no overt differences in the size and shape of the entire cerebellum and more specifically, the vestibulocerebellum in the knockout vs. the wild type mouse (Supplementary Fig. 5).

**Figure 3:**
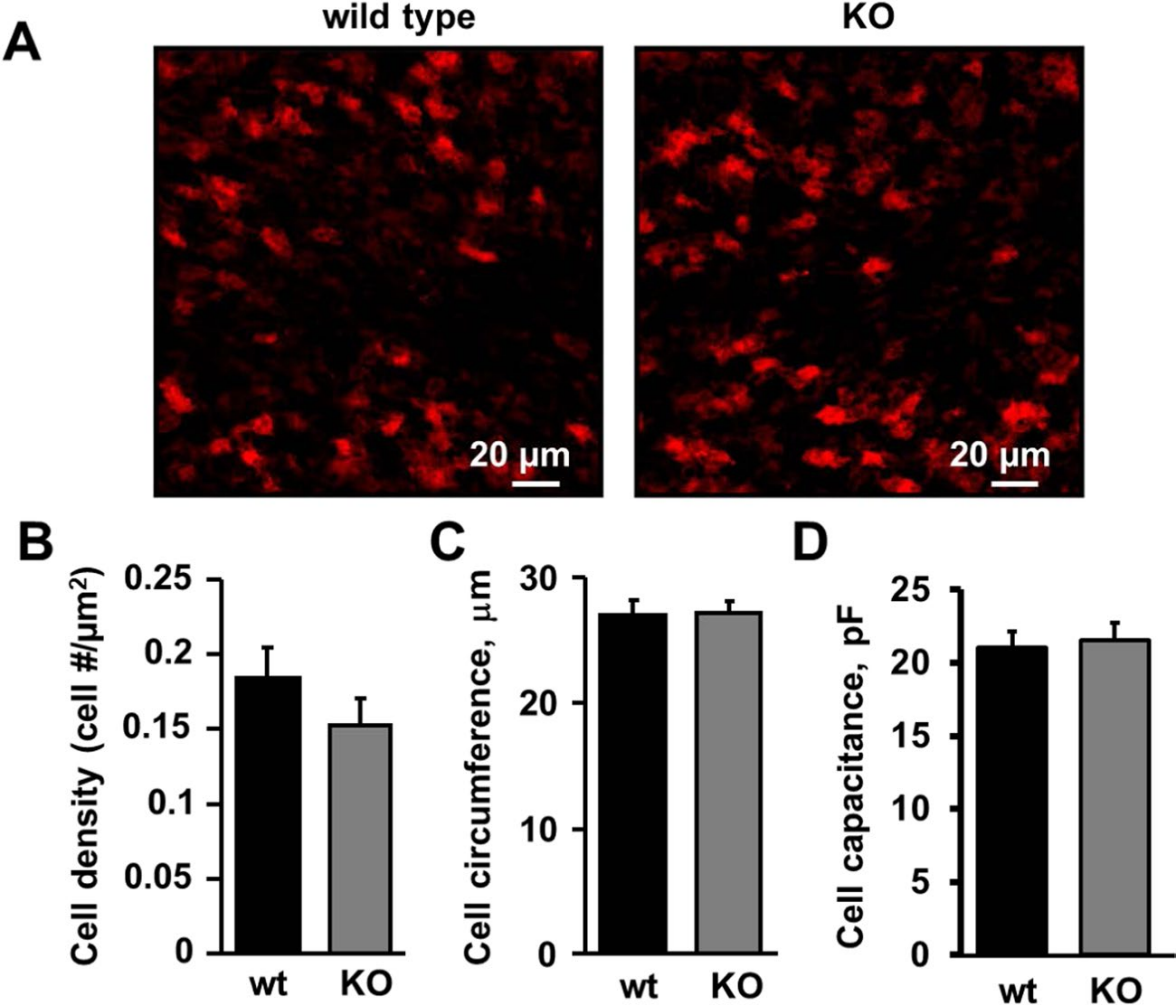
Deletion of Asic5 does not affect type II UBC density, size or capacitance. (**A**) Representative Z-projected confocal images of mGluR1α positive type II UBCs immuno-labeled with anti-mGluR1α (red) antibody in lobule X of cerebellar cryosections prepared from control (left) and Asic5 KO (right) mice. Summary graphs of mGluR1α- and GFP-positive type II UBC mean densities (**B**) and circumferences (**C**), respectively, in cerebellar sections from wild type (black bars) and Asic5 KO (gray bars) mice. Summary data for type II UBC density from 9 (3 mice, mean age P60) control and 15 (3 mice mean age P60) knockout cryosections, respectively. Summary data for type II UBC circumference from 53 littermate (5 different cryosectons from 2 different animals) and 58 knockout (7 different cryosections from 2 different animals) type II UBCs. (**D**) Summary graph of GFP-positive type II UBC capacitance in cerebellar sections from wild type (black bars; n = 9, 7 slices, 4 animals, P17 age) and Asic5 KO (gray bars; n = 9, 8 slices, 4 animals, P16.8 age) mice. No significant difference was found between wild type and Asic5 KO for any of these measurements using a two tailed unpaired *t*-test.

Balance and motor coordination defects in the Asic5 KO mouse could be associated with changes in the number of type II UBCs and/or the morphology of these interneurons as a result of Asic5 deletion. To explore these possibilities, we compared the mean cell densities and the circumferences of type II UBCs in lobules IX and X of the cerebellum in wild type versus KO mice. For these experiments, type II UBCs in midsagittal cerebellar slices were identified by immuno-staining against mGluR1α. Shown in Fig. 3A are representative fluorescence micrographs of such staining in wild type (left) and Asic5 KO (right) mice. The dependent summary graphs in 3B and C demonstrate that type II UBC density (0.18 ± 0.02 vs. 0.15 ± 0.02 cell #/μm^2^) and circumference (27.0 ± 1.3 vs. 27.3 ± 0.9 μm), respectively, are not significantly different in wild type vs. Asic5 KO mice. The mean capacitance of GFP-positive type II UBCs in addition was assessed. As shown in 3D, type II UBC capacitance is not different in KO type II UBCs (21.54 ± 1.19 pF) vs. those in wild type (21.01 ± 1.16 pF). These results are consistent with impaired balance and coordination in the Asic5 KO mouse being independent of changes in the number, size and gross morphology of type II UBCs suggesting that functional changes in these interneurons may underpin this change in behavior.

### Deletion of Asic5 reduces the inherent excitability of type II UBCs

**Figure 4:**
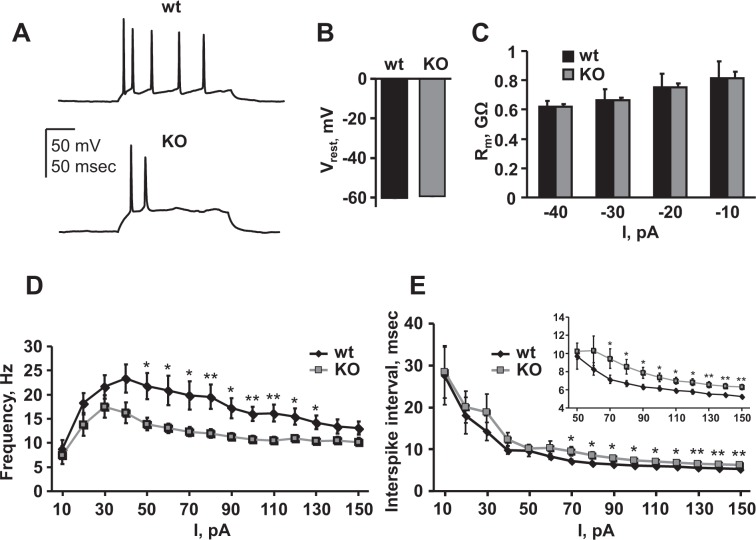
Asic5 influences the inherent excitability of type II UBCs. (**A**) Representative traces of typical action potential trains in current-clamped type II UBCs in vestibulocerebellar slices from wild type (top) and Asic5 KO (bottom) mice evoked by 200 msec 40 pA supra-threshold current injections. Summary graphs of mean resting membrane potentials (**B**) and membrane resistances (**C**) in current-clamped type II UBCs from wild type (black bars, n = 32, 15–20 slices, 5 animals, mean age P19) and Asic5 KO (gray bars, n = 28, 12–15 slices, 4 animals, mean age P18) mice. No value is significantly different. Summary graphs of mean firing frequencies (**D**) and interspike intervals (**E**) for action potentials evoked by current injections in current-clamped type II UBCs in vestibulocerebellar slices from wild type (black circles, n = 32, 5 animals) and Asic5 KO (gray boxes, n = 28, 4 animals) mice. Inset in E shows an enlarged area for significantly different values for interspike intervals evoked by current injections greater than 65 pA. Significantly different at *P < 0.05 and **P < 0.01 with *t*-test.

To begin revealing the role of Asic5 in type II UBCs, and to better understand how loss of this ion channel’s activity in type II UBCs may influence broader cerebellar function and animal behavior, we compared the electrophysiological properties of these interneurons in wild type and KO mice. Shown in Fig. 4A are representative voltage traces from typical current clamped GFP-positive type II UBCs in brain slices from wild type (top) and Asic5 KO (bottom) mice. The observed bursting firing pattern in response to supra-threshold current injections is a hallmark of type II UBCs and has been previously described^6,29,34^. Summary results from such experiments, as shown in Fig. 4B–E, demonstrated that mean resting membrane potentials (Fig. 4B) and input resistances (Fig. 4C) were not different in type II UBCs of wild type vs. KO mice, but that firing frequencies (Fig. 4D) and interspike intervals (Fig. 4E) were significantly reduced and increased, respectively, in type II UBCs of the Asic5 KO vs. wild type mouse. The simplest interpretation of these observations is that the increase in interspike interval resulting from deletion of Asic5 caused a dependent reduction in firing frequency in type II UBCs of the knockout mouse. Consequently, deletion of Asic5 reduces the inherent excitability of type II UBCs.

### Deletion of Asic5 slows the depolarization rate of action potentials in type II UBCs

**Figure 5:**
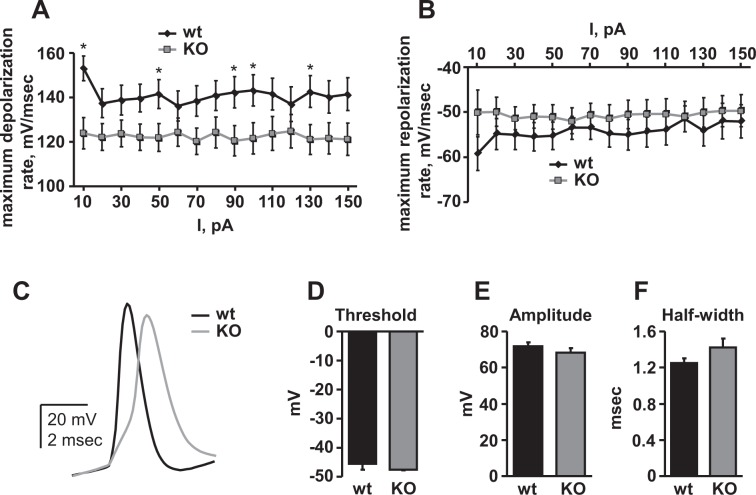
Deletion of Asic5 slows the maximum depolarization rate of type II UBCs. Summary graphs of maximum depolarization (**A**) and repolarization (**B**) rates of first action potentials evoked by current injections in current-clamped wild type (n = 32, 15–20 slices, 5 animals, mean age P19) and Asic5 KO (n = 28, 12–15 slices, 4 animals, mean age P18) type II UBCs. *Significantly different using a *t*-test. (**C**) Overlay of representative first action potentials from current-clamped type II UBCs injected with 70 pA from wild type (black) and Asic5 KO (gray) mice. Summary graphs of mean thresholds (**D**), amplitudes (**E**), and half-widths (**F**) for first action potentials in type II UBCs evoked by 10 pA supra-threshold current injections in wild type (n = 13, 5 animals) and Asic5 KO (n = 8, 3 animals) mice. No value is significantly different.

The observed reduction in firing frequency of type II UBCs from Asic5 KO animals could be the result of a change to the shape of the action potential. To test this possibility, we analyzed the first action potentials evoked by current injections ranging from 10 to 150 pA, and compared the results for type II UBCs from wild type and Asic5 KO mice. The corresponding summary graphs in Fig. 5 demonstrate that the maximum depolarization rate of action potentials (Fig. 5A) in type II UBCs of the KO mouse are markedly slowed compared to those from wild type mice, but the maximum repolarization rate of action potentials (Fig. 5B) is little affected by deletion of Asic5. Figure 5C shows representative overlays of first action potentials from wild type and KO type II UBCs elicited by a 70 pA supra-threshold current injection highlighting this difference in depolarization rate.

Further analysis of first action potentials in type II UBCs from wild type and Asic5 KO mice revealed, as shown in Fig. 5D–F, that action potential thresholds (Fig. 5D) and amplitudes (Fig. 5E) were unaffected by deletion of Asic5, but that action potential half-widths (Fig. 5F), while not significantly different, trended towards being broader in type II UBCs from KO mice vs. those from wild type mice. The latter likely is reflective of the slower maximum depolarization rate of action potentials in type II UBCs of the KO mouse. Together these observations suggest that Asic5 contributes to the upstroke of the action potential in type II UBCs, either through direct contribution to the depolarizing inward current or through secondary effects on voltage-gated sodium channels.

### Glutamate-evoked slow outward K^+^ currents are decreased in type II UBCs of the Asic5 KO mouse

**Figure 6:**
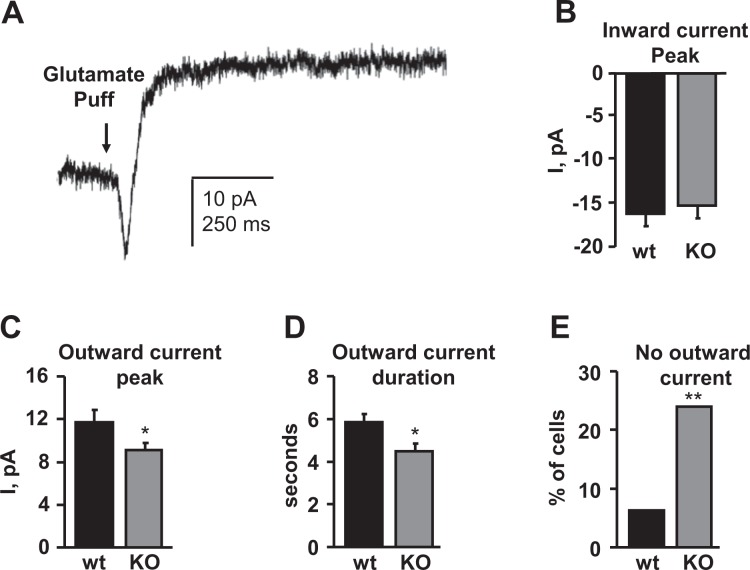
The magnitude and presence of hyperpolarizing, slow outward K^+^ currents in type II UBCs treated with glutamate are decreased in the Asic5 KO mice. (**A**) Shown here is mean current as a function of time evoked by a train of 10 mM glutamate puffs from a compilation of raw current traces from a representative voltage-clamped type II UBC in a typical vestibulocerebellum brain slice from a wild type mouse. Currents evoked by application of glutamate were biphasic in type II UBCs with a fast depolarizing inward current seen initially followed by a slower developing hyperpolarizing outward current. Summary graphs of glutamate-evoked inward (**B**) and outward (**C**) current amplitudes, and outward current durations (**D**) in voltage-clamped type II UBCs from wild type (n = 32, 15–25 slices, 5 animals, mean age P20) and Asic5 KO (n = 55, 21–35 slices, 7 animals, mean age P19) mice. Summary data from experiments similar to that in A. *Significantly different compared to wild type with a *t*-test. (**E**) Percentage of type II UBCs from wild type (n = 32, 5 animals) and Asic5 KO (n = 55, 7 animals) mice having no outward currents in response to treatment with 10 mM glutamate. Data from experiments are similar to A. *Significantly different compared to wild type with a χ^2^-test.

As expected, reported previously^9^ and shown here in Fig. 6A, glutamate quickly evokes an excitatory, depolarizing inward current in type II UBCs within a brain slice by stimulating AMPAR and mGluR1α receptors. This excitatory inward current is rapidly followed by a hyperpolarizing outward K^+^ current. Such responses to glutamate enable type II UBCs to function as “ON” cells where inward currents are pro-excitatory and outward K^+^ currents resist excitability. As summarized in Fig. 6B–E, glutamate-sensitive outward but not inward currents are significantly decreased in type II UBCs of the KO vs. control mouse. When observed, the peak magnitude (11.7 ± 1.1 vs. 9.1 ± 0.6 pA, Fig. 6C) and duration (5.8 ± 0.3 vs. 4.5 ± 0.3 sec, Fig. 5D) of outward K^+^ currents evoked by glutamate were reduced in type II UBCs of Asic5 KO vs. control mice. Moreover, as shown in Fig. 6E, only 76% of type II UBCs in the KO had measurable outward K^+^ currents following glutamate treatment as compared to 94% of these cells in wild type mice. As reported above, the intrinsic excitability of type II UBCs is decreased in the Asic5 KO mouse. This suggests the decrease in outward K^+^ currents could be a compensatory response to normalize cell excitability.

### Deletion of Asic5 reduces spontaneous firing but increases burst firing in response to glutamate

**Figure 7:**
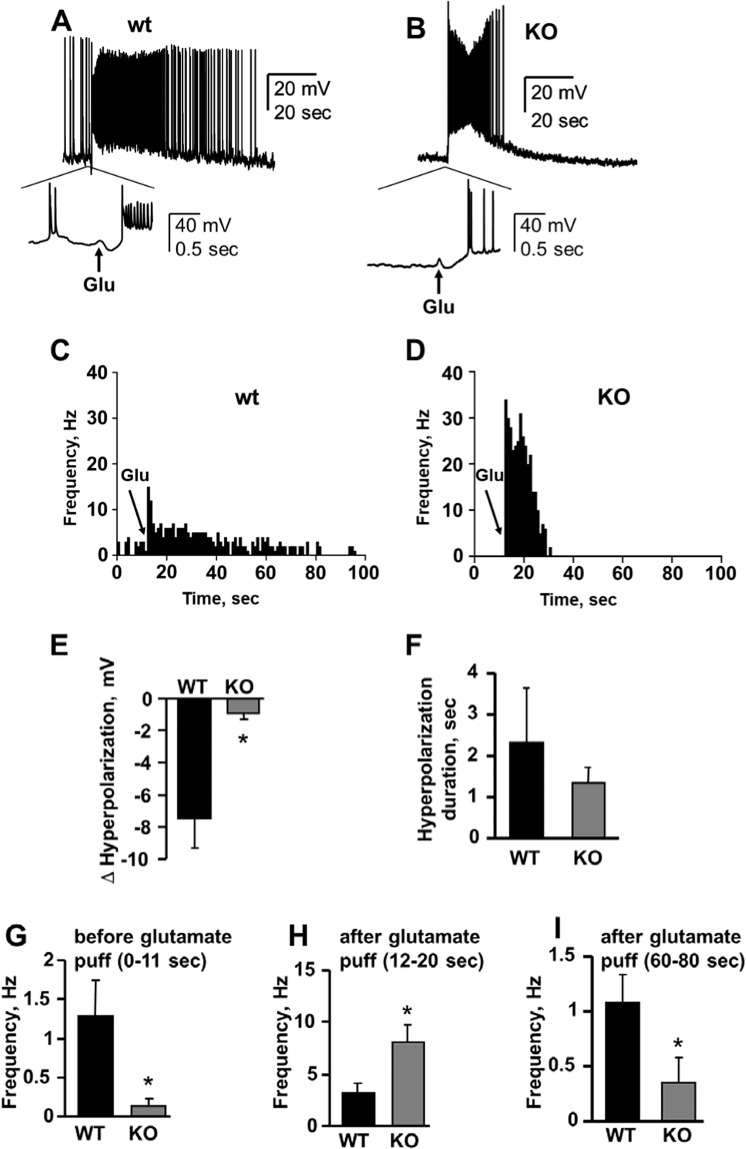
Deletion of Asic5 reduces spontaneous firing but elevates glutamate-evoked burst firing frequency in type II UBCs. Representative gap-free traces of spontaneous and evoked action potential trains in typical current-clamped type II UBCs before and after treatment with 10 mM glutamate (arrow) in vestibulocerebellar slices from wild type (**A**; n = 9, 6 slices, 4 animals, mean age P16.5) and Asic5 KO (**B**; n = 10, 6 slices, 3 animals, mean age P16)) mice. The period during initial exposure to glutamate is shown at an expanded scale below. Representative instantaneous firing frequency histograms over the duration of such experiments from the same cells as in **A** and **B** (**C**,**D**, respectively). The point at which glutamate was added is shown with an arrow. Summary graphs of membrane potential (**E**) and duration (**F**) of hyperpolarization following treatment with glutamate in wild type (black bars) and Asic5 KO (gray bars) type II UBCs. Data from experiments identical to that shown in A and B. Significantly different at *P < 0.05 with *t*-test. Summary graphs of mean firing frequency for spontaneous (**G**) and burst (**H**) action potential firing, and then spontaneous firing again (**I**) before and after exposure to glutamate for wild type (black bars) and Asic5 KO (gray bars) type II UBCs. Summarized data from experiments identical to those shown in (**A**,**B**). Spontaneous firing prior to exposure to glutamate was quantified during the 0–11 second window, burst firing after exposure to glutamate was quantified during the 12–20 second window, and the return to spontaneous firing quantified over the 60–80 second window. Significantly different at *P < 0.05 with *t*-test.

Type II UBCs have two modes of firing: slow spontaneous firing; and higher frequency burst firing in response to glutamate, which is preceded by a brief hyperpolarization^5,6,9,10,34^. By comparison, type I UBCs typically have a higher spontaneous firing frequency with glutamate suppressing action potential spiking. To determine if the decreases in inherent excitability and glutamate-sensitive outward K^+^ currents noted above affect the firing of spontaneous and glutamate-evoked action potentials, we quantified spontaneous and burst firing before and after treatment with glutamate in current-clamped type II UBCs in brain slices of control and Asic5 KO mice. Figure 7A,B show representative voltage traces from current-clamped wild type and KO type II UBCs before and after addition of glutamate. As shown by these traces, and also in the representative instantaneous frequency histograms shown in 7 C and D for action potential firing in wild type and KO type II UBCs, respectively, and as summarized in 7G-I, spontaneous firing (Fig. 7G) in untreated type II UBCs from wild type mice is significantly greater than that in Asic5 KO mice, 1.29 ± 0.46 vs. 0.14 ± 0.09 Hz. However, as shown in 7E and F, the hyperpolarization following glutamate application in Asic5 type II UBCs is reduced in magnitude (−7.48 ± 1.83 vs. −0.92 ± 0.31 mV) and duration (2.3 ± 1.3 vs 1.3 ± 0.4 sec) compared to wild type cells, likely due to a reduction in glutamate-sensitive outward K^+^ currents in these cells (see Fig. 6). As a result, burst firing following glutamate application begins from a less hyperpolarized membrane potential in type II UBCs from the Asic5 KO. The consequence of this is reported in Fig. 6H: Burst firing frequency following stimulation with glutamate is elevated in type II UBCs from Asic5 KO as compared to wild type mice, 8.05 ± 1.70 vs 3.17 ± 0.95 Hz, respectively. Within 60–90 seconds after exposure to glutamate, as shown in Fig. 7I, spiking has slowed returning to normal where spontaneous firing frequency is again higher in wild type as compared to KO type II UBCs, 1.08 ± 0.25 vs. 0.35 ± 0.23 Hz, respectively.

## Discussion

The primary conclusions supported by this work are that Asic5 serves a key function in type II UBCs, influencing intrinsic excitability and the firing of spontaneous action potentials, as well as, glutamate-sensitive burst firing; and that deletion of Asic5 causes discoordination. There likely is a cause and effect relation between these two observations such that type II UBCs serve a key role in the vestibulocerebellum, and their dysfunction here due to abnormal Asic5 activity causes a mild form of ataxia. However, this latter possible mechanistic relation is tempered, to a degree, by the global nature of the knockout model used in these studies. Contributions made by vestibulocerebellum dysfunction to the discoordination observed in the Asic5 KO mouse could possibly be interacting with or confounded by changes in muscle function and anxiety behaviors, amongst other variables and tissue functions not investigated here. Nonetheless, the current observations offer rich evidence in support of the idea, as proposed first by others^5,9–11,34^, that type II UBCs serve as a type of “ON” cell where their normal spontaneous spiking, glutamate-sensitive phase delay and burst firing play essential roles in appropriate temporal coding during sensory-motor integration by the vestibulocerebellum.

As reported previously by us^29^, and briefly recapiculated here in Supplementary Fig. 1, using an *Asic5* reporter mouse, Asic5 is chiefly expressed in the brain in type II UBCs. Here, we evolved this reporter mouse, as supported by results in Supplementary Fig. 2, into a global Asic5 knockout model to begin testing the function of this channel in type II UBCs and to determine if dysfunction of type II UBCs could contribute to discoordination. During study of this knockout mouse, we also were able to elaborate the cellular and electrical mechanisms underpinning type II UBC dysfunction in this model.

Results in Figs. 1 and 2 are consistent with dysfunction of type II UBCs contributing to cerebellar ataxia, due primarily to a vestibulocerebellum defect in the Asic5 KO mouse model. We argue this because deletion of Asic5 causes discoordination, Asic5 is restrictively expressed in type II UBCs^29^, type II UBCs are chiefly expressed in the vestibulocerebellum^4,6^, and because cerebellar dysfunction is widely recognized as being capable of causing ataxia. However, type II UBCs are also expressed in the DCN, and to a lesser degree in other parts of the cerebellum and brain, and broader body tissues. Thus, the global nature of this knockout model cannot fully exclude possible interactions or confounding effects contributing to the observed discoordiantion resulting from loss of Asic5 activity in other tissues.

Because it was tested here, as reported in Supplementary Fig. 4, we can exclude anxiety-like behaviors, though, from contributing to the phenotype of the Asic5 KO mouse. Moreover, we note that the ataxic phenotype observed in the Asic5 KO mouse, as supported by results in Figs. 1 and 2, and Supplementary Figs. 3 and 4, is mild and apparent at an early age. Perhaps this milder phenotype reflects the fact that only one type of ion channel in a small subset of interneurons expressed only in a restricted manner in the vestibulocerebellum of the broader cerebellum are moderately electrically defective (see below) in the Asic5 KO model as compared to other more severe models, to include *Moonwalker* and *reeler* mice^14–16,35^, which have global ablation and/or loss of function of many types of cortical principal cells, to include widespread loss of Purkinje cells in the cerebellum. Moreover, that the mild ataxic phenotype of the Asic5 KO mouse does not worsen with age is consistent with a functional defect at the cellular level rather than widespread loss of neurons in the cerebellum (discussed more below).

As supported by results in Fig. 3 and Supplementary Fig. 5, ataxia in the Asic5 KO mouse is unlikely to be caused by broad changes in the abundance, locale and morphology of type II UBCs or gross changes in vestibulocerebellum development, as is thought to be the case for the *reeler* and *Moonwalker* mouse models^14–16,35^, indicating that changes in the electrical properties of type II UBCs are most likely at fault here. Of note in the current study is that we observed electrical differences in type II UBCs of the knockout compared to control animals at an age where the ataxic phenotype is already apparent. Results in Figs. 4 and 7 showing that type II UBCs in the Asic5 KO mouse have decreased intrinsic excitability, fire fewer spontaneous action potentials but at a higher frequency following glutamate stimulation are consistent with changes in electrical properties of type II UBCs contributing to ataxia in this model. Such a mechanism would share some similarity to that possibly underpinning ataxia in the *Moonwalker* and *Weaver* mouse models^3,14^; though because of generalized cerebellar neuronal wasting and misplacement it has been difficult to assess if changes in cerebellar neuronal activity strongly contributes to the ataxic phenotype of these earlier models. *Moonwalker* mice have a gain of function mutation in *TrpC3*, which is expressed in type II UBCs^14,16^. Abnormal Ca^++^ signaling due to enhanced TrpC3 activity is expected to affect type II UBC electrical activity and thus, likely may be an early contributor to ataxia that precedes the loss of type II UBCs in this mouse. Similarly, abnormal electrical activity of type II UBCs could potentially be contributory to ataxia in the *Weaver* mouse for a missense substitution in *Girk2* resulting in loss of function of this key ion channel is the primary gene defect in this model^36^. Like in many neurons, Girk2 contributes strongly to hyperpolarization in UBCs, and rebound from this hyperpolarization resulting from AMPAR recovery from desensitization is thought to underpin the delayed burst firing in glutamate-stimulated type II UBCs^11^. Loss of Girk2 activity, thus, could be expected to change the electrical properties of type II UBCs compromising temporal signal processing in cerebellar circuits containing these interneurons. The degree to which such potential changes in type II UBCs contributes to ataxia versus broader generalized neuronal death, which is a hallmark of the *Weaver* mouse, is unknown. In comparison, the current results offer evidence linking a specific change in type II UBC electrical activity to the discoordination observed in the Asic5 KO mouse. Further tying a change in electrical activity to ataxia in the Asic5 KO mouse are the findings in Figs. 4, 5 and 7 demonstrating that a slowed maximum depolarization rate and consequent increase in interspike interval may be the cellular causes of the decrease in intrinsic excitability and spontaneous firing observed in type II UBCs from the Asic5 KO mouse.

Results in Fig. 6 show that the magnitude and duration of glutamate-sensitive hyperpolarizing K^+^ currents in type II UBCs of the Asic5 KO mouse are decreased. We believe that this is a compensatory response because lessening of a hyperpolarizing current should favor an increase in excitability and perhaps spontaneous spiking. Clearly, as documented in Figs. 4 and 7, the opposite was observed. Furthermore, the Asic5 channel primarily passes an inward Na^+^ current. As such, loss of this channel is more consistent with a decrease in excitability and a compensatory down-regulation of K^+^ current.

Consequences of possible compensatory decreases in glutamate-sensitive K^+^ currents, as supported by data in Fig. 7, are that the duration and the magnitude of hyperpolarization preceding the burst of action potentials following glutamate stimulation trend shorter, and is significantly more depolarized, respectively, in type II UBCs of the Asic5 KO mouse. Moreover, firing from this more depolarized potential drives burst firing to a higher frequency in type II UBCs from the Asic5 KO mouse as compared to those in normal mice.

The ultimate result of these electrical differences in type II UBCs of the Asic5 KO mouse are phase shifts and changes in spontaneous and burst firing frequencies that likely result in inappropriate temporal coding and resolution in the target signal in vestibulocerebellar networks containing this interneuron. As consistent with results reported here, such a defect likely is capable of driving impaired motor coordination and abnormal balance.

## Methods

### Animal care and use

All animal use and welfare in these studies adhered to the National Institutes of Health Guide for the Care and Use of Laboratory Animals following protocols reviewed and approved by the Institutional Animal Care and Use Committee of the University of Texas Health at San Antonio. Mice were housed and cared for by Laboratory Animal Resources at the University of Texas Health at San Antonio, which is fully accredited by the Association for Assessment and Accreditation of Laboratory Animal Care, and licensed by the United States Department of Agriculture. ARRIVE guidelines^37^ were followed for reporting experiments and results involving animals.

For behavioral studies, trained weanlings and mature male Asic5 knockout and littermate control mice were used. Only trained mature mice were used to assess gait; whereas, untrained, niave weanlings were used for the open-field test. All mice were maintained at room temperature with normal 12 hr. light/dark cycles and free access to water and chow and housed socially with littermates and peers. The primary endpoints for experiments involving mice in these studies were assessment of motor balance and coordination, gait, general activity and anxiety-like behaviors using the accelerating rotarod performance test, a balance beam walking assay, a gait analysis and an open-field test. Mice were trained for these tests, except for the open-field test which they were niave to, three times over a three day period prior to testing. Following humane euthanasia, neonatal and weanling (P14–25) male mice also served as a source of cerebellar slices for assessment of UBC activity with patch clamp electrophysiology.

### The Asic5 knockout mouse

Initial development of the Asic5 knockout mouse has been described previously^29^. In brief, the founder line containing the *Asic5*^*tm2a(KOMP)Wtsi*^ allele (from the Knockout Mouse Project (KOMP) repository; Univ. Cal., Davis) was created by injecting JM8A3.N1 (Agouti) embryonic stem cells positive for homologous recombination into blastocysts from a B6D2F1 x B57BL/6 donor^38^. Congenic germline transmitting mice homozygous for the *Asic5*^*tm2a(KOMP)Wtsi*^ allele (genotyping of this founder mouse described previously in Fig. 1 of ref. ^29^) were crossed to homozygous B6.129S4-*Gt(ROSA)26Sor*^*tm1(FLP1)Dym*^/RainJ mice (The Jackson Laboratory; stock 009086) to remove the *frt*-flanked SA-βgeo-pA gene trapping cassette in this allele via FLP-mediated recombination to produce a line containing *loxP*-flanked *Asic5* (*Accn5*) with conditional potential. The resulting offspring were crossed to B6.C-Tg(CMV-cre)1Cgn/J mice (The Jackson Laboratory; stock 006054) producing the whole animal Asic5 knockout mouse through deletion of exon 3 of the *Asic5* gene via Cre-mediated recombination. Homozygous Asic5 knockout mice then were crossed with mice harboring the *Tg(Grp-EGFP)DV197Gsat* transgene. The latter mice were generated by the GENSAT project and have restrictive expression of GFP primarily in mGluR1α positive type II UBCs^6,39,40^. We obtained these mice with permission from Drs. M. Martina and E. Mugnaini (Northwestern University, Evantson IL). The resulting *Asic5*+/− mice, which also expresses GFP in type II UBCs, were backcrossed to B57BL/6 mice for four generations and inbred back to homozygosity for the KO allele and GFP.

Genotyping with a standard polymerase chain reaction (PCR) was used to confirm FLP-FRT and Cre-LoxP recombination, and generation of floxed and KO mice. For genotyping reactions, mouse genomic DNA was extracted from tail samples using the DirectPCR (Tail) Lysis Reagent (Viagen) following the manufacturer’s instructions. PCR was performed with genomic DNA using the MangoMix PCR Kit (Bioline). The allele specific primers used in these reactions were for wild type the forward primer, 5′-AGCTGACTGCTGGCTGGTTATGTG-3′, and the reverse primer, 5′-TTTGGCTTAACTGACCATAAAGGC-3′, which produce an expected product of 351 bp; for floxed and KO, the common forward primer, 5′-AGGCGCATAACGATACCACGATATC-3′, plus the floxed specific reverse primer, 5′-CTATTCCGCCTACTGCGACTATAGA-3′, which produce an expected product of 217 bp; and the KO specific reverse primer, 5′-GATTGCCCTGATGTCCTTATGTCC-3′, which produce an expected product of 284 bp.

### Assessment of balance, coordination, motor performance and gait

Motor balance and coordination of control and Asic5 KO mice were assessed following standard procedures^41,42^ using a Rota-Rod Treadmill (Med Associates Inc., Fairfax, VT) that had a 32 mm diameter rod. An accelerating test protocol was used and included starting at 4 RPM with an increase to 40 RPM over a 300 second period at an acceleration of 7.2 RPM per minute. All mice were placed on the rod before rotation was initiated. Following 10 seconds of static walking at 4 RPM, the rod was accelerated with fall times recorded using a platform timer stop switch. In some cases, inversion times were noted instead of fall times. Inversion was when a mouse stopped walking and gripped the rod firmly to passively somersault around the rod two or more times in succession. (For mature control and knockout mice approximately 1/3 of the animals inverted; whereas, for weanlings 1/11 and 1/4 of control and knockout mice inverted, respectively. Mice that inverted almost uniformly held on for the duration of the test, releasing only after stoppage of the rod.) Mice were trained on this equipment for three days prior to experimentation. The performance of an individual mouse was assessed in triplicate on consecutive runs separated by a 10 minute start-to-start rest and recovery period. The mean fall time for each individual mouse was then averaged (from the triplicate measurements) with the cohort mean run-times being the average of these averages. For rotarod experiments, each mouse was tested in triplicate only once. To quantify fall speeds in the acceleration mode, the following equation was used:

$${\rm{RPM}}=[({\rm{end}}\,{\rm{speed}}-{\rm{start}}\,{\rm{speed}})/300\,{\rm{seconds}}]\times ({\rm{seconds}}\,{\rm{run}})+{\rm{start}}\,{\rm{speed}}$$

Run-times exceeding 300 seconds, after which no acceleration occurred, were taken as 300 sec for fall speed calculations.

Fine motor coordination and balance was assessed using standard procedures^41–43^ on a static, elevated horizontal balance beam. The beam apparatus was a 1 cm diameter wood beam that was 70 cm in length and raised 15 cm from the bench top. A cage was placed at the end of the beam as a safety platform. Mice were trained on this apparatus for three consecutive days prior to experimentation. Beam crossing to the safety platform for each mouse was measured in triplicate with a 10 minute rest interval between measurements. To ease analysis, mouse performance was recorded with a fixed video camera set on a tripod. The time, total number of steps taken to complete the beam and the number of slips/step for each trial (one completion of the apparatus) were averaged for each mouse. The mean time to completion, steps taken, and number of slips per step plus standard error of the mean was calculated by quantifying the average of these averages where each mouse was used only once.

Gait analysis was performed following standard protocols^30,44^. In brief, footprint patterns were analyzed using nontoxic ink to paint fore and hind limb paws and white paper lining the runway of an open-topped tunnel (50 cm long × 10 cm wide × 10 cm high) ending in a closed-topped safe haven. Resulting footprint patterns from mice walking down the runway were then analyzed for distance between each stride (limb stride), overlap of each step (step overlap), variability in the base width between left and right paws (base width), and foot angle around a linear axis (foot angle).

### Assessment of anxiety-like and exploratory behavior, and activity in an open-field

The open-field test used in the current study to examine anxiety-like behvaior has been previously described^31–33^. In brief, niave weangling mice were placed into a 56 × 56 cm white box with movement monitored over a 5 minute trial period under dim lighting. A video tracking system using a DVC model HDV-6045 HD video camera combined with EthoVision XT software (Noldus Informaiton Technology Inc., Leesburg, VA) was used to quantify locomotor activity and time spent in the central zone (central 26 × 26 cm region) and border areas (the 10 cm closest to the wall) of the box.

### Immunohistochemistry

Immunohistochemistry studies followed previously published protocols^29^. In brief, deeply anesthetized mice were perfused transcardially with saline and then ice cold 2% paraformaldehyde in PBS. Perfusion fixed murine brains were isolated and subsequently fixed an additional two hours at 4 °C with 2% paraformaldehyde. Brains were then cryoprotected with 30% glucose at 4 °C for 24–48 hours. Sagittal cryosections (40 µm thick) through the vermis of dissected cerebellums were prepared on a freezing-stage microtome and subsequently mounted on charged superfrost-plus glass slides. Sections were blocked for 30 minutes at room temperature with 3% BSA in PBS containing 0.1% Triton X-100 and then incubated with the primary antibody, rabbit anti-β-galactosidase (1∶5,000; MP Biomedicals), mouse anti-GFP (1:5,000; Millipore) or mouse anti-mGluR1α (1:200; BD Biosciences), at 4 °C overnight. Sections then were exposed to secondary antibody and DAPI as normal. The anti-rabbit and anti-mouse secondary antibodies used in these studies were conjugated to either Alexa 488 or Alexa 568 (Life Technologies). Fluorescence images of cerebellar sections and UBCs were acquired using an IX81/FV-1000 confocal microscope (Olympus).

UBC density was measured in sagittal cerebellar slices taken from the vermis and labelled with anti-mGluR1α antibody. Labelled type II UBCs were identified by eye and counted in 212 × 212 μm z-projected confocal images taken from lobules IXc and X in wild type or Asic5 KO tissue. The circumference of UBCs were measured in cerebellar sections in mice labelled with anti-GFP antibody using ImageJ (NIH) software. A post hoc power analysis (2 means: 2-sample, 2-sided equality; α = 0.05, power (1-β) = 0.8) revealed that the sample sizes, means and SEMs for circumferences used in the current studies would identify a change that is greater than 5% at a 95% confidence level.

### Brain slice preparation

Parasagittal brain slices were prepared fresh from the cerebella of Asic5 KO and control mice. Mice were deeply anesthetized with isoflurane before rapid dissection of the cerebellum. The cerebellum was immediately placed in ice-cold (0–4 °C), oxygenated (95% O_2_ and 5% CO_2_) artificial cerebrospinal fluid (aCSF) containing (in mM): 119 NaCl, 26.2 NaHCO_3_, 2.5 KCl, 1.0 NaH_2_PO_4_, 11 glucose, 2 CaCl_2_, and 1.3 MgCl_2_. Parasagittal slices (300 μm thick) were cut from the cerebellar vermis using a vibratome (Leica Biosystems, Buffalo Grove, IL, USA) and transferred to a recovery chamber filled with oxygenated aCSF. Slices were maintained at 34 °C for 30 min after which they were allowed to return to room temperature and kept under continuous oxygenation until transferred to the recording chamber.

### Analysis of cerebellar morphology

Cerebella were weighed prior to slicing. Parasagittal slices (300 μm thick) were prepared as above from the cerebellar vermis, 6 slices per cerebellum, using a vibratome (Leica Biosystems, Buffalo Grove, IL, USA) and transferred to a recovery chamber filled with oxygenated aCSF. Slices were imaged using a Nikon D3300 camera with a macro lens. Circumference of the slice, total length of the Purkinje cell layer, surface length of the vestibullocerebellum (lobules 9 and 10), and length of the Purkinje cell layer in the vestibulocerebellum were then quantified for each image using ImageJ (NIH) sofware.

### Electrophysiology

During recordings, slices were superfused with room temperature aCSF at a flow rate of ~2 mL/min using a recirculating pump (Cole-Parmer, Vernon Hills, IL, USA), except where noted. For patch-clamp analysis, the cells in the vestibulocerebellar granular layer of lobules IX and X were visualized with Dodt contrast videomicroscopy^45^ using a 60x water-immersion objective on an upright SliceScope Pro microscope (Scientifica). Type II UBCs, positive for GFP expression, were visually identified using a GFP filter and an epifluorescence LED light source (470 nm, CoolLED). Seals were formed on these interneurons using borosilicate glass capillaries (3–6 MΩ) (Sutter Instruments) filled with an internal pipette solution containing (in mM): 137 K-gluconate, 2KCl, 4 MgCl_2,_ 10 HEPES (pH 7.3–7.4), 5 EGTA, 4 Na-ATP, and 0.5 Na-GTP at an osmolarity of 280–300 mOsm/L. Whole-cell current- and voltage-clamp recordings were made using a Multiclamp 700B amplifier (Molecular Devices, Sunnyvale, CA, USA), filtered at 5 kHz and digitized at 50 kHz. Data were collected using pCLAMP software (Axon Instruments) and analyzed with IGOR Pro software (Wavemetrics, Portland, OR).

For current-clamp experiments, current was injected into the cells to maintain a resting membrane potential of −60 mV, when necessary. Cells with a resting membrane potential above −55 mV were not included in further analysis. Action potentials from type II UBCs were recorded using the whole-cell current-clamp configuration in the presence of 100 μM picrotoxin (PTX, Abcam) and 10 μM NBQX (Sigma) in the external bath solution to block GABA_A_ and AMPA receptors, respectively. Action potentials were evoked with 200-ms depolarizing current injections through the patch pipette. Interspike intervals were measured for the first two evoked action potentials per current injection. The first action potential evoked per current injection was used to determine action potential threshold, amplitude, half-width, and maximal rates of depolarization and repolarization. Membrane resistance was determined from step hyperpolarizing current injections (−40 to −10 pA). Cell capacitance was measured as normal by calculating the area of the capacitive transient during a hyperpolarizing step in voltage-clamped type II UBCs and dividing by the amplitude of the step (5 mV).

Glutamate-mediated currents and action potentials were evoked by 100 ms pressure application (PDES-02DX; NPI electronic, Tamm, Germany) of 10 mM glutamate (Sigma) from a perfusion pipette at the surface of the slice. During such experiments, the external bath solution contained 100 μM PTX and was not recirculated. Prior to analysis of evoked inward and outward currents, the acquired traces where smoothed using a ten point boxcar algorithm. Peak inward and outward currents were quantified between 100–800 and 1000–10,000 msec, respectively, following glutamate treatment. The duration of the outward current was defined as the time period required to return to baseline. In current clamp experiments, the spontaneous firing rate was measured between 0 to 11 seconds, the glutamate evoked hyperpolarization was measured as the peak hyperpolarization within 1 second of glutamate application, the glutamate evoked firing rate was measured between 12 to 20 seconds, and the recovery firing rate was measured between 60 to 80 seconds.

### Equipment and settings

Images of PCR genotyping gels, including those in Supplementary Fig. 2, were captured as JPGs on an iPhone using the SmartDoc (Accuris Instr.) system equipped with a 590 nm bandpass filter. Images were imported into PowerPoint 2016 for further processing to include black/white color inversion, and adjustment of brightness and contrast equally across the entire image.

### Statistics

Summarized data are reported as mean ± SEM. Summarized data compared with either a *t*-test or chi-squared test as noted in each figure legend. A *P* ≤ 0.05 was considered statistically significant.

## Supplementary information

Supplementary figures.
