# Supplementary material for: Cerebellar Ataxia Caused by Type II Unipolar Brush Cell Dysfunction in the Asic5 Knockout Mouse
Source: Sci Rep. 2020 Feb 7;10:2168. doi: 10.1038/s41598-020-58901-y (PMC7005805; doi:10.1038/s41598-020-58901-y)
Supplement: Supplementary file 1 — Supplementary figures. [file 41598_2020_58901_MOESM1_ESM.docx]

**Cerebellar Ataxia Caused by Type II Unipolar Brush Cell Dysfunction in the Asic5 Knockout Mouse**

Tabita Kreko-Pierce^1,2^, Nina Boiko^1,2^, Donald G. Harbidge^3^, Daniel C. Marcus^3^, James D. Stockand^1,*^ and Jason R. Pugh^1^

^1^Department of Cellular and Integrative Physiology

University of Texas Health Science Center at San Antonio

San Antonio, TX 78299

^2^These authors contributed equally to this work.

^3^Department of Anatomy and Physiology

Kansas State University

Manhattan, KS 66506

^*^Correspond with J.D. Stockand at

stockand@uthscsa.edu

210-567-4333

**Supplementary Figures.**


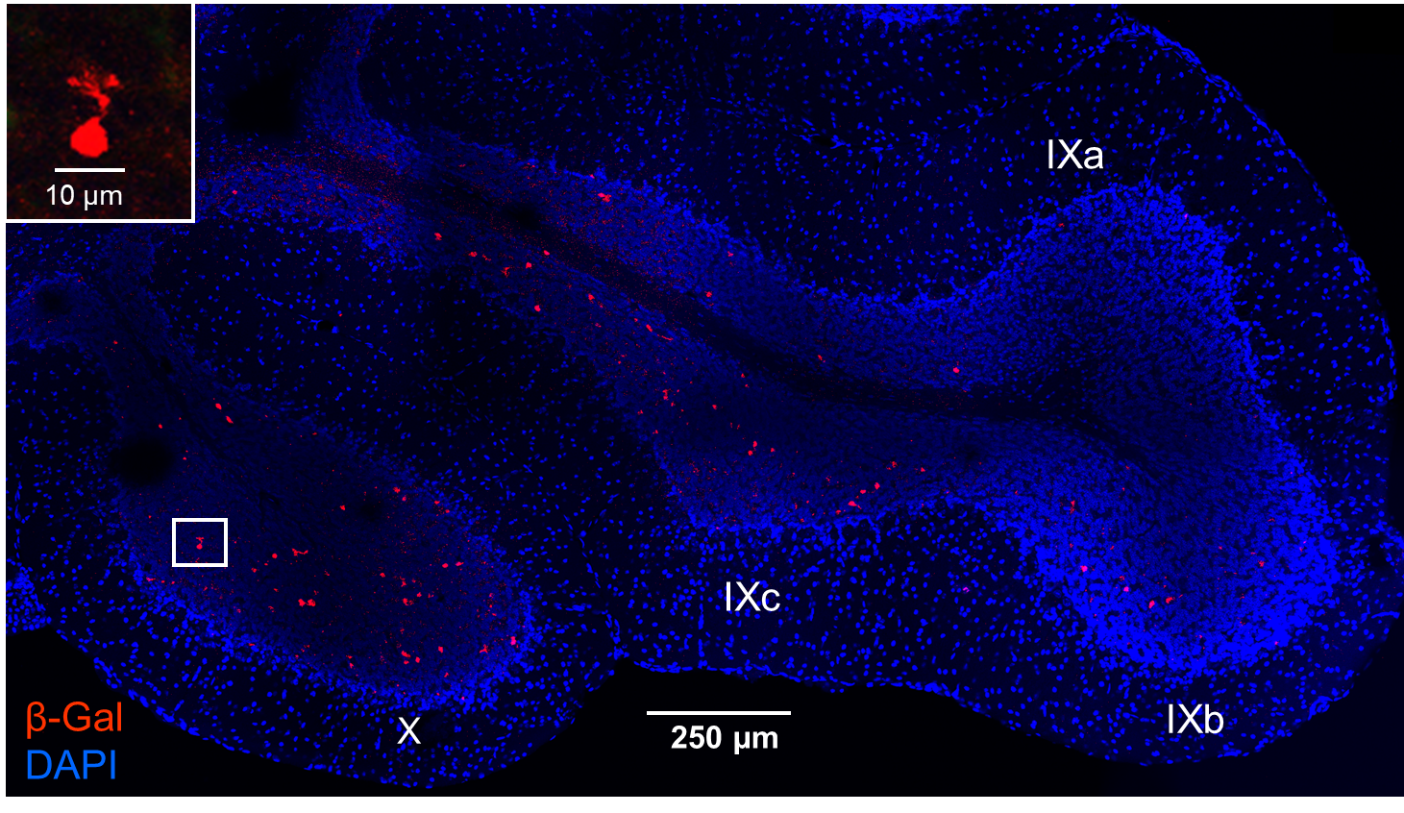


**Supplementary Figure 1. Asic5 is restrictively expressed in type II unipolar brush cells.** Shown is a representative fluorescence micrograph of lobules IX and X in a typical midsagittal section of the cerebellum from the Asic5^tm2a(KOMP)Wtsi^ reporter mouse stained with anti-β-Gal (red) and DAPI (blue). An area of lobule X, as outlined by the white box, containing a typical granular layer interneuron positive for β-Gal expression in the reporter mouse is shown, at top left, at a magnified scale.

**
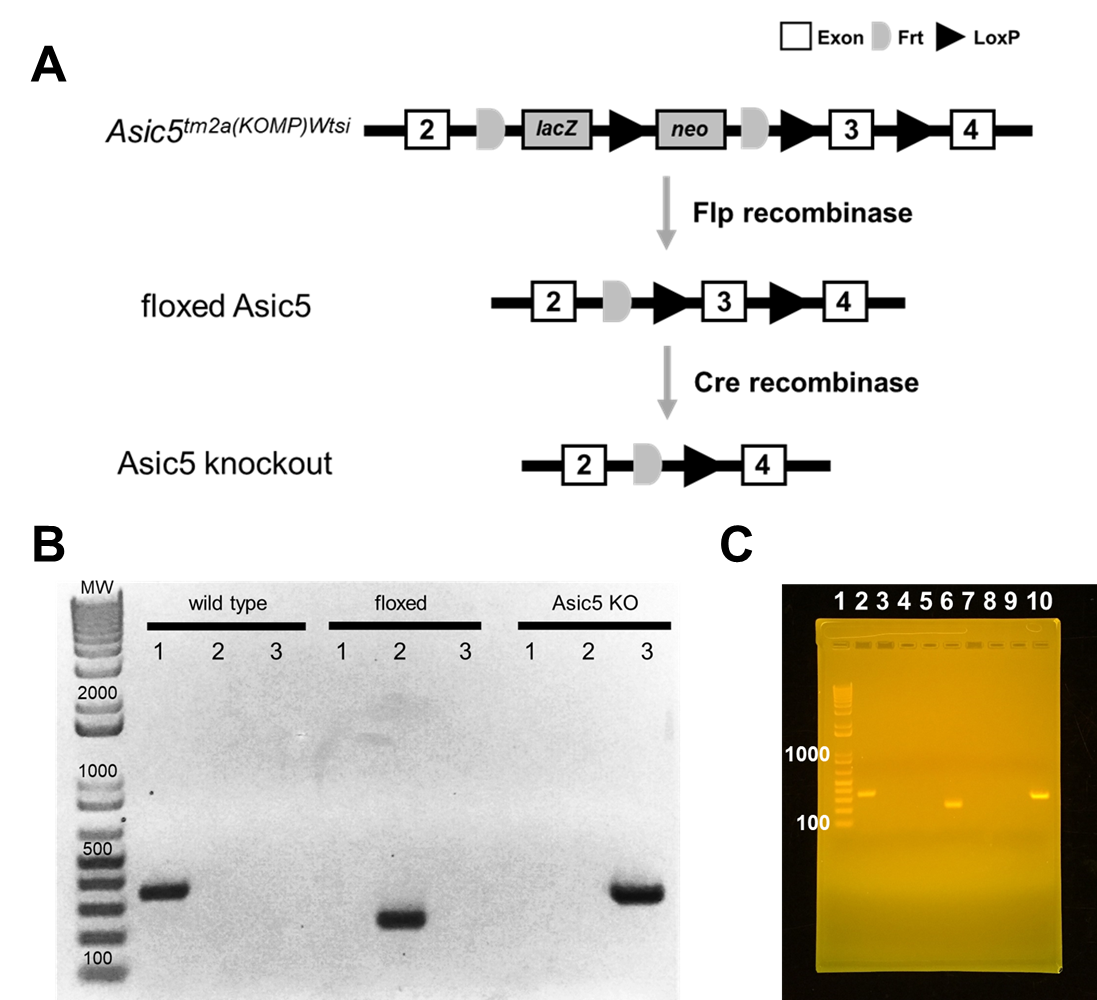
**

**Supplementary Figure 2. Generation of the Asic5 knockout mouse. A.** Shown is a simplified schematic representation of the creation of the Asic5 KO mouse from the Asic5^tm2a(KOMP)Wtsi^ reporter mouse. This scheme shows the founder *Asic5^tm2a(KOMP)Wtsi^* allele containing the LacZ-Neo gene trapping cassette (gray boxes) between exons 2 and 3 (white boxes), and the adjacent Frt (gray half-moons) and LoxP (black arrowheads) sites used to create the dependent floxed *Asic5*, and *Asic5* knockout alleles. **B.** Shown is an inverted image of a representative gel containing typical products from genotyping reactions of homozygous wild type (351 bp), floxed (217 bp), and Asic5 KO (284 bp) mice using allele specific primers. The original image (shown in C) was converted to a gray scale and inverted to create this figure to increase clarity. This image also has been cropped and minor adjustments made to the contrast and sharpness to further increase clarity. **C.** The original, untouched image of the full-length, uncropped gel shown in supplementary figure 2B. Shown is a representative gel containing typical products from genotyping reactions of homozygous wild type (lanes 2-4 from the left), floxed (lanes 5-7 from the left), and Asic5 KO (lanes 8-10 from the left) mice using allele specific primers to identify the wild type (lanes 2, 5 & 8), floxed (lanes 3, 6 & 9) and Asic5 KO (lanes 4, 7 & 10) alleles in these animals. The first lane of this gel includes 1 Kb Plus DNA Ladder (Invitrogen) molecular weight markers of twenty bands - 100 (lowest), 200, 300, 400, 500, 650, 850, 1,000, 1,650 bp, and 2-12 Kb in 1 Kb increments with 12 Kb being the top band.

**A**

**
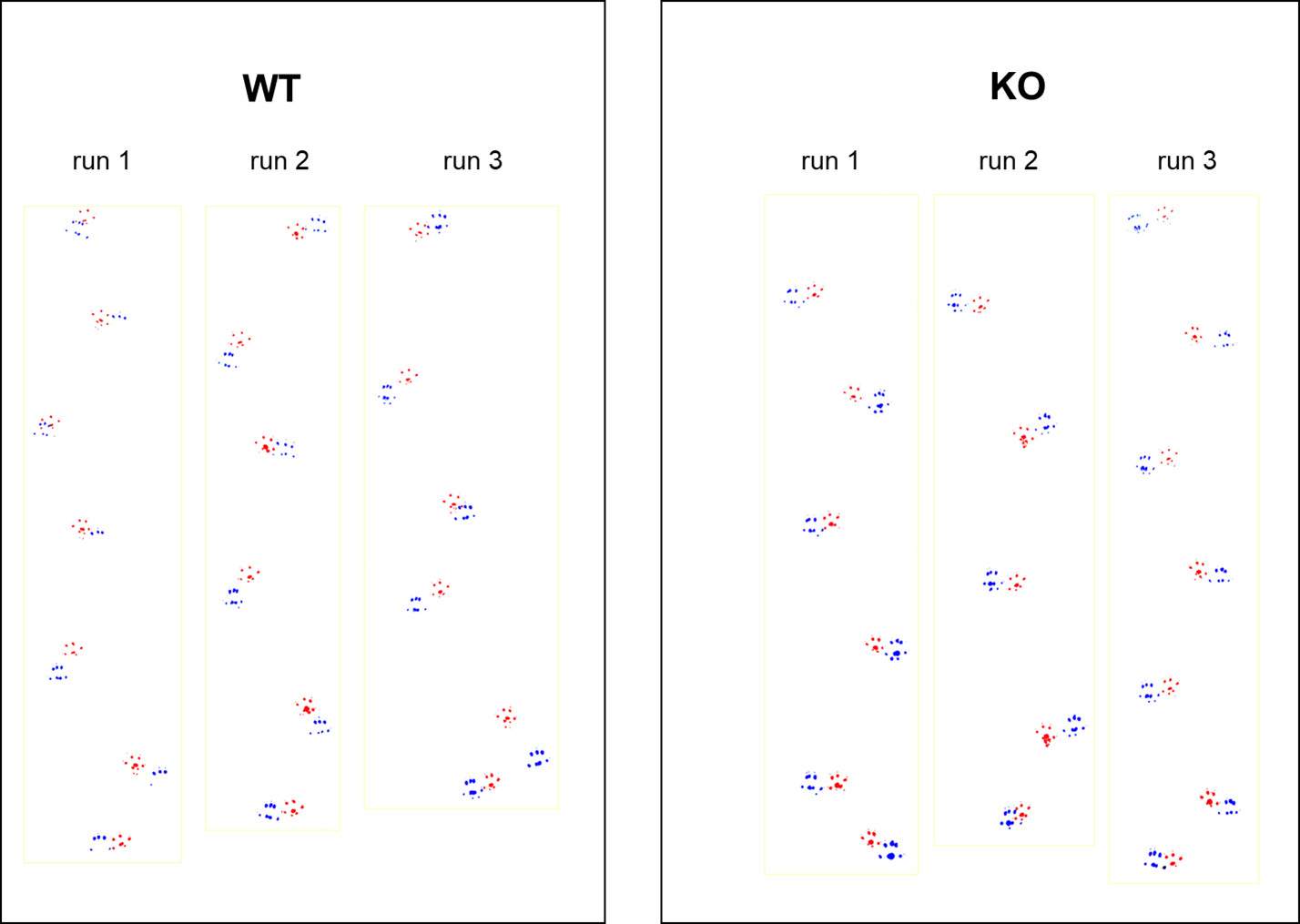
**

**B**

| **gait variable quantified** | **side (left/right)** | **wild type** | | | **Asic5 KO^1^** | | |
| --- | --- | --- | --- | --- | --- | --- | --- |
|  |  | **MEAN** | **SEM** | **n** | **MEAN** | **SEM** | **n** |
| limb stride, front^2^ | left | 3.03 | 0.05 | 5 | 3.27 | 0.06 | 5 |
|  | right | 3.11 | 0.05 | 5 | 3.31 | 0.04 | 5 |
| limb stride, hind | left | 2.98 | 0.07 | 5 | 3.17 | 0.06 | 5 |
|  | right | 2.80 | 0.04 | 5 | 3.25 | 0.06 | 5 |
| step overlap | left | 0.34 | 0.01 | 5 | 0.38 | 0.02 | 5 |
|  | right | 0.41 | 0.01 | 5 | 0.40 | 0.03 | 5 |
| base width, front | left | 0.53 | 0.03 | 5 | 0.53 | 0.02 | 5 |
|  | right | 0.47 | 0.02 | 5 | 0.21 | 0.03 | 5 |
| base width, hind | left | 1.23 | 0.01 | 5 | 1.12 | 0.01 | 5 |
|  | right | 1.34 | 0.13 | 5 | 1.07 | 0.02 | 5 |
| foot angle, front | left | 2.71 | 1.27 | 5 | -1.68 | 1.03 | 5 |
|  | right | -3.50 | 1.30 | 5 | -1.04 | 1.13 | 5 |
| foot angle, hind | left | -1.10 | 1.18 | 5 | -2.00 | 1.34 | 5 |
|  | right | 5.46 | 1.59 | 5 | 0.47 | 1.81 | 5 |

^1^No variable significantly different between wt and KO. ^2^Limb stride, step overlap and base width in cm; foot angle in degrees.

**Supplementary Figure 3. Quantification of wild type and Asic5 KO gait.** Footprint patterns were quantified following standard protocols using nontoxic ink, red for forelimbs and blue for hind limbs. Patterns were quantified from each mouse in triplicate with 5 distinct mature male mice used per group, wild type (85.4 ± 1.5 days old, 26.3 ± 0.4 g) and ASIC5 KO (86.6 ± 1.5 days, 25.2 ± 0.4 g). **A.** Representative striding for a wild type (left) and Asic5 KO (right) mouse in triplicate. **B.** Table containing summary results of gait analysis for wild type and Asic5 KO mice. No significant difference noted.


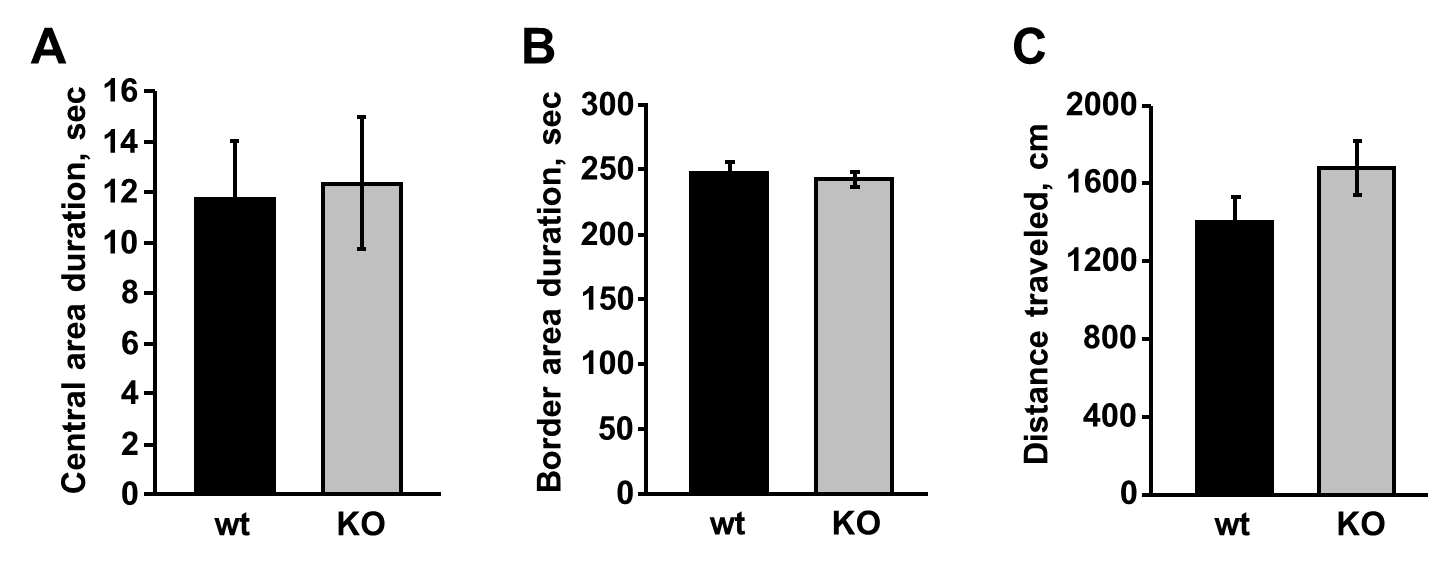
**Supplementary Figure 4. Mice that lack Asic5 have normal behavior in an open-field test.** Summary graphs of mean time spent in central (**A**) and border (**B**) areas, and total distance traveled per unit time (5 minutes; **C**) for niave wild type (black bars) and Asic5 KO (gray bars) mice. Summary data from 13 different male weanling littermate (age - 28.2 ± 1.2 days, wt - 14.3 ±0.8 g) and 14 different male weanling Asic5 KO (age 27.8 ± 1.8 days, wt - 14.3 ±0.8 g) mice. No significant difference noted with a two tailed unpaired *t*-test.


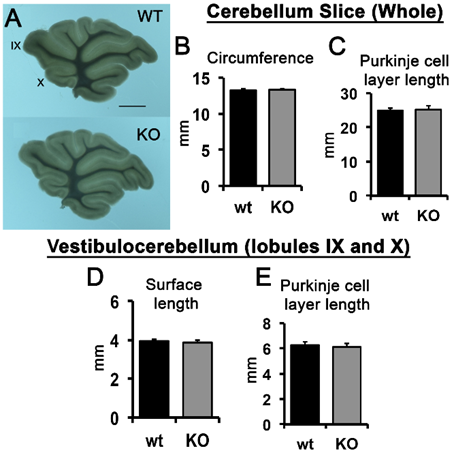


**Supplementary Figure 5. Deletion of Asic5 does not affect the size of the cerebellum or vestibulocerebellum. A.** Representative images of cerebellar slices (300 μm thick) taken from the vermis of wild type (top) and Asic5 KO (bottom) mice at postnatal day 16. Scale bar = 1 mm. Summary graphs of whole cerebellar slice circumference **(B)** and Purkinje cell layer length **(C)** from wild type (black bars; 12 slices from 2 animals) and Asic5 KO (gray bars; 18 slices from 3 animals) mice. Summary graphs of vestibulocerebellum (lobules IX and X) surface length **(D)** and Purkinje cell layer length **(E)** from wild type (black bars) and Asic5 KO (gray bars) mice. No significant difference was found between wild type and Asic5 KO for any of these measurements using a two-tailed unpaired t-test.
